# Dynamical model of aperiodic locomotor activity effects on mouse core body temperature removes transient perturbations from longitudinal temperature signals

**DOI:** 10.1038/s41598-025-31953-8

**Published:** 2026-01-06

**Authors:** Jamison H. Burks, Benjamin L. Smarr

**Affiliations:** 1 Shiu Chen - Gene Lay Department of Bioengineering, University of California, San Diego, USA; 2 Halıcıoğlu Data Science Institute, University of California, San Diego, USA

**Keywords:** Neuroscience, Physiology, Zoology

## Abstract

Mammalian temperature changes across time due to multiple endogenous and exogenous factors including circadian rhythms, hormonal changes, and locomotor activity. These multiple factors make it difficult to disentangle each of their effects to understand their independent contributions. This is especially problematic due to the relatively high-amplitude, aperiodic heating effects of locomotor activity on core body temperature. These heating effects, combined with innate cooling effects back to core body temperature steady state, mean that locomotor activity can contribute apparent power to both circadian and ultradian rhythms in observed temperature data. We propose that the effect from locomotor activity to core body temperature is not simply the linear addition of circadian and ultradian oscillations, but rather a heating effect that can be offset by a cooling effect dependent on core temperature displacement from resting temperature. Since these effects appear to contribute power to independent rhythms in spectral analysis, in this work we develop an interpretable, parsimonious mathematical model of murine core body temperature that removes them in the time-domain. The model only depends on the initial observed core body temperature as well as minute-level locomotor activity data, making it robust to aperiodic mouse activity. We show that coefficients obtained after fitting the model to each mouse return physiologically relevant differences between sexes, as well as reflect directional changes within female mice between their non-estrous and estrous temperature data. We believe this work should be of use to researchers interested in how core body temperature dynamics change in response to experimental interventions, especially if locomotor activity may be affected as well.

**Supplementary Information:**

The online version contains supplementary material available at 10.1038/s41598-025-31953-8.

## Introduction

Mammalian temperature changes across time. These changes are caused by (non-exhaustively) endogenous processes^1–3^ (e.g., circadian rhythms, hormonal changes, etc.), exogenous processes^4,5^ (e.g. ambient temperature, eating behaviors, etc.), and locomotor activity (LA)^6,7^. Different changes in temperature may therefore indicate changes in disparate underlying processes – but this is difficult to study due to large amplitude temperature changes driven by LA. We hypothesize that given paired signals of temperature and LA, we can develop a model that accounts for the impact of LA on temperature, as well as temperature’s return to steady state amplitudes. This model could be used to remove those LA- and cooling-associated temperature changes. The leftover temperature changes should then be those associated with other endogenous processes. To test this hypothesis, we must make some initial assumptions about heating effects of LA and cooling dynamics in murine core body temperature (CBT). To test whether the residual CBT in fact seems biologically meaningful, we should define some expectations of the patterns that may be found within these endogenous processes (e.g., circadian and ultradian rhythms).

It seems likely that there is not a simple linear relationship between greater LA and greater CBT in mammalian physiology^8^, as mammals have a fairly narrow range of acceptable temperatures, and many mechanisms to avoid overheating^9,10^. Assuming CBT is at a resting value before any LA, we presume that activating muscles from rest engages more metabolic mechanisms. These metabolic mechanisms likely introduce a state-dependent heating effect such that at greater amplitudes of CBT the change from any given amount of LA should be smaller compared to when CBT amplitudes are closer to the resting CBT amplitude^11^. Then, given a lack (or decrease) in LA, there should be a cooling effect with an effect proportional to the CBT displacement from its resting CBT amplitude^5^. Therefore, we hypothesize that a state-dependent relationship exists between changes in LA and CBT. A model that explicitly accounts for the causal effect of LA on CBT would theoretically leave residuals which reveal the non-LA dynamics in CBT regulation. If temporal structure still exists in the residuals after the modeled effects have been differenced out, then that structure may contain information relating to other covariate-dependent dynamics such as circadian and ultradian rhythms.

**Fig. 1:**
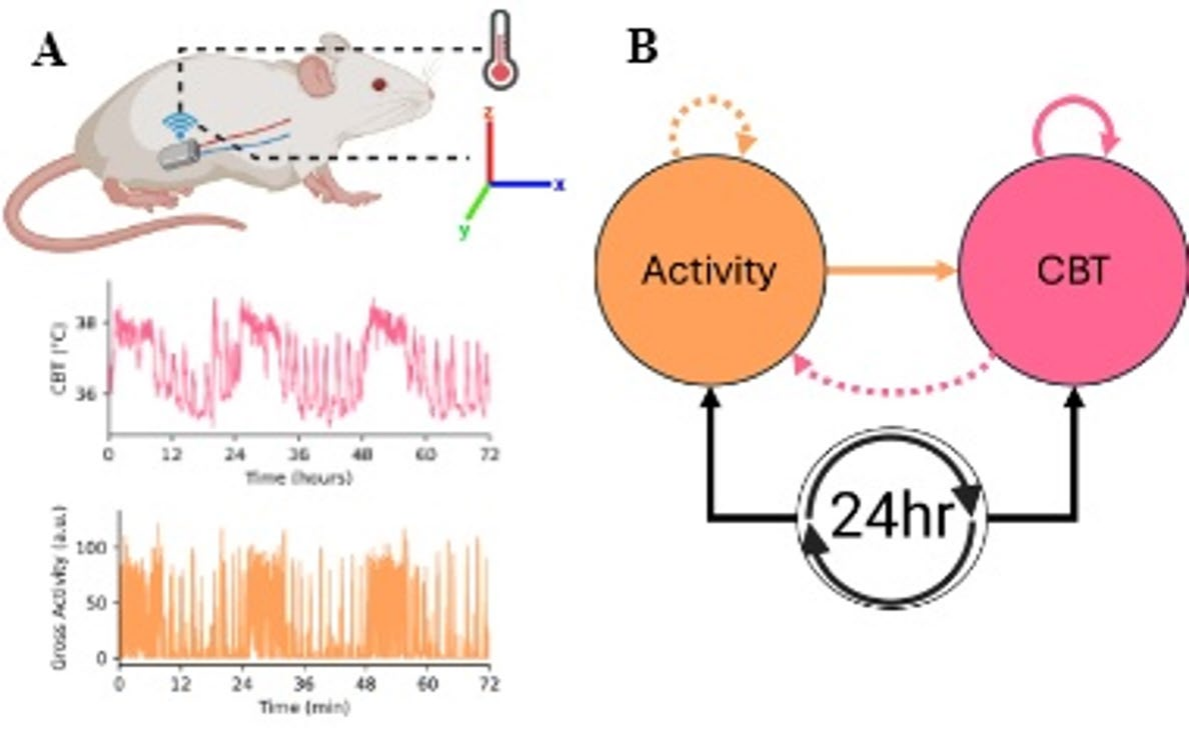
A. 3-day example of Core Body Temperature (CBT, pink) and Locomotor Activity (LA, orange) data sampled every minute from a male mouse. **B**. Visual representation of a proposed network. Solid arrows indicate the relationships being investigated in this work. Dashed arrows indicate relationships that are hypothesized to exist but are not investigated in this work.

Circadian and ultradian rhythms are known to affect both LA^12,13^ and core body temperature (CBT)^2,3^. These are hypothesized to both be influenced in part through dynamical control of outputs from the suprachiasmatic nucleus^14–16^. Because we assume that the relationships between LA and CBT are state-dependent, we cannot rely on classical statistics or linear spectral methods (e.g., Fourier, Lomb-Scargle, etc.) to disentangle them. Continuous and/or quasiperiodic increases in LA can therefore lead to continuous and/or quasiperiodic increases in CBT, which ultimately makes it challenging to pull out components of CBT resulting from non-LA processes, such as pulses of hormones^17^, changes in the vascular lumen radius^18^, or even the CBT set point itself^19^. More specifically, since CBT amplitudes decay towards a steady state after perturbations induced by LA, LA may in fact contribute substantial spectral power to *both circadian and ultradian bands* in CBT (Fig. 1). Sustained LA during a dark period followed by inactivity during a light period would appear as power in the 24-hour band of a periodogram. Intermittent, aperiodic LA during the light period would appear as power in the ultradian band. This contributed spectral power may be independent from underlying hormonal circadian and ultradian effects on CBT.

In this work, we utilize LA signals sampled minutely from 13 male and 13 female mice in a constant temperature environment to develop a candidate state-space model that predicts 14 days of minute-level CBT data. The model uses only the *initial observed value* of CBT for each mouse as well as the minute-level actigraphy data to ensure stability at long time horizons. It is dependent only on *state variables* (CBT and LA) and not time as a variable, making it robust to nonstationary LA. The model was designed by hand, inspired by the observed dynamics, in order to ensure model generalizability, parsimony, and interpretability. The coefficients of the models fit to each mouse were evaluated for sex- and estrous-specific differences in dynamical state variables such as the minimum temperature, LA effect on CBT, and the innate heat loss of CBT. We compared this model to a proposed model by Weinert and Waterhouse, 1998^20^ that performs a linear statistical fit between LA and CBT. This prior model assumes no temporal interdependence between CBT data and thus we propose does not account for the inherence cooling process of CBT back to a minimum value.

The residuals after differencing out the predictions for each mouse were compared across male (M), female non-estrous (NE), and female estrous (E) days to identify time-dependent or state-dependent differences in dynamics over the course of the median day of residual error (the errors after removing the state-dependent effects). We continue to observe circadian and ultradian power in CBT dynamics even after accounting for the deterministic heating of LA and cooling of CBT, indicating that this proposed mathematical model can potentially be used in interventional studies where a researcher is interested in dynamical changes to thermoregulatory control while not preventing mice from engaging in active lifestyles. While it is certainly possible (and arguably the gold standard) to compare mouse states using Fourier analysis, the innovation of this work is to highlight that apparent cyclicity can arise out of instantaneous causal interactions in LA and CBT processes, thus exaggerating an underlying circadian oscillation and making its estimation sensitive to differences in mouse activity.

## Methods

### Data gathering

Data published previously from Smarr, 2017^21^ were re-analyzed, with no new animal experiments conducted (raw data can be found in the Supplementary Material). Briefly, data were previously generated using 13 male and 13 female 8–12-week-old BALB/c mice using 1-min resolution recordings of CBT and LA. CBT and LA were gathered using a G2 minimitter (Starr Life Sciences Co., Oakmont, PA), implanted (several weeks prior to start of recording) in the intraperitoneal cavity and secured to the inside of the abdominal wall to maintain consistency. Mice were kept in a LD 12:12 L: D photocycle with ad libitum access to water and chow. Light onset and offset occurred at 0600 and 1800 h, respectively. Humidity and temperature were held constant at 40% and 21 °C, respectively. For female mice, days of estrous were determined in Smarr, 2017 based on 4-day periods in which CBT had prolonged elevation as suggested in Sanchez-Alavez, 2010^22^.

Animals were not handled or otherwise disturbed during the 14-day period of data assessed here, other than weekly cage changes. The original data had been cleaned to replace any missing values with the within-mouse median of the 14-day period of data collected. For this work, the median-interpolated values were replaced with the linear interpolation between the preceding and following points.

### Model fitting

The Python library *SciPy* was used to fit each model. Specifically, the *scipy.optimize.minimize* function was used to minimize the following cost function:

1 $$\:J\left(y,\:\widehat{y},\:w\right)=\sum_{t=1}^{N}({y}_{t}-{\widehat{y}}_{t}{)}^{2}+\lambda\:\sum_{j=1}^{M}{w}_{j}^{2}$$

Where $\:{y}_{i}$ is the actual value for the $\:i$-th training example, $\:{\widehat{y}}_{i}$ is the predicted value for the $\:i$-th training example, $\:{w}_{j}$ is the $\:j$-th weight in the model, and $\:\lambda\:$ is the regularization parameter, which controls the strength of the $\:{L}_{2}$ penalty. $\:{L}_{2}$ Regularization was utilized to prevent parameters from becoming too large as well as to minimize potential collinearity between the parameters.

The set of predicted values, $\:\widehat{Y}=({\widehat{y}}_{1},\:{\widehat{y}}_{2},\dots\:,{\widehat{y}}_{N-1},{\widehat{y}}_{N})$, are created from the following models:

2 $$\:{\widehat{y}}_{t}=m\sum_{t}^{t+\tau\:}{A}_{t}+b$$

3 $$\:{\widehat{y}}_{t}={\widehat{y}}_{t-1}+\frac{{\Delta\:}{\widehat{y}}_{t-1}}{{\Delta\:}t},\:\:\:\frac{{\Delta\:}{\widehat{y}}_{t-1}}{{\Delta\:}t}={a}_{1}{A}_{t-1}+{a}_{2}\left({\widehat{y}}_{t-1}-{T}_{min}\right),\:\:\:{\widehat{y}}_{1}={T}_{1}$$

$\:{T}_{t}$ is the measured CBT at time index $\:t$. $\:m$ and $\:b$ are the slope and intercept effects of a linear fit of LA to $\:T$ under the Weinert and Waterhouse, 1998 model^6^; $\:\tau\:$ is the time lag needed to reach a maximum correlation to $\:T$ (for this dataset the median optimal lag for all mice was 10 min). $\:{a}_{1}$ and $\:{a}_{2}$ are the coefficients for the LA input ($\:{A}_{t-1})$ and the CBT difference between estimated temperature ($\:{\widehat{y}}_{t-1}$) and minimum temperature ($\:{T}_{min}$), respectively.

During the model fitting process for Eq. 2, only the initial observed temperature ($\:{T}_{1}$) and time-indexed $\:{A}_{t-1}$ values were used to begin integrating the estimated $\:\widehat{Y}$ timeseries, thus generating a prediction horizon of 20,159 min of CBT from LA data. Each mouse had models fit to their data, thus creating errors and parameters that were specific to each mouse. Residuals were based on the predicted 14-days of CBT data against the observed 14-days of CBT data.

### Statistical comparison of different model performances

Root mean squared errors (RMSE) from each model were separated by sex. These sex-separated errors were then first compared across models using Kruskal-Wallis tests by ranks. Upon rejection of the null hypothesis that the samples in all groups originate from the same distribution ($\:\alpha\:$ = 0.05), post-hoc Dunn’s tests were performed between all models’ error distributions, and false discovery rate was corrected with the Benjamini-Hochberg adjustment.

### Statistical comparison of errors and coefficients from male, non-estrous, and estrous models

Errors and coefficients from male mice models were compared to model errors and coefficients from non-estrous and estrous models using Mann-Whitney U tests. Errors and coefficients from non-estrous models and estrous models from within-female mouse models were compared with Wilcoxon Signed-Rank tests since they were fit from different days from the same mouse ($\:\alpha\:$ = 0.05).

### Aggregation and comparison of temperature residuals after model fit

Residuals were separated based on if they came from male, non-estrous, or estrous days. The median day of residuals for each of the male, non-estrous, and estrous days were generated by aligning each lights-off/lights-on pair by the start of lights off. Since the CBT trajectories were all aligned by time (1440 samples), then the median day from each mouse’s CBT trajectory of residuals is the median error at each minute of the day. There were 14 days of male residuals for each male mouse, 9 days of non-estrous days for each female mouse, and 4 days of estrous days within those same female mice. These median days were combined for each group (male, non-estrous, and estrous) to be used for time-of-day comparisons. The sum of the residuals between 2 h and 4 h post-lights off were calculated for each median male and female non-estrous day to compare CBT residual differences soon after active time. The sum of the residuals between 0 h and 6 h post-lights on were calculated for each median female estrous day to be compared to the time-matched residuals for male and female non-estrous days to compare the CBT residual differences driven by estrous.

### Welch’s periodogram of median days and comparison to white noise periodogram

The periodograms for each male mouse and non-estrous/estrous residuals were calculated using Welch’s method (*scipy.signal.welch*) using 48 h of samples (2880 samples sampled once per minute) with an overlap of 24 h. Linear detrending was performed for each window prior to calculating the periodogram. Since each periodogram was calculated for each mouse, each of those longitudinal residuals had 13 periodograms (1 for each mouse’s male or non-estrous/estrous day). The null hypothesis was that the residuals were generated from Gaussian white noise. In a periodogram, which averages spectral power over multiple windows, the spectral power density approaches a small value (depending on random noise amplitude) above zero as more windows are included. The Gaussian white noise was generated by randomly shuffling the population residuals for each category (male, non-estrous, and estrous) 1000 times. On each shuffle, Welch’s periodogram was calculated with the same parameters described for the original residuals. The 95th percentile of those Gaussian white noise periodograms was used as the boundary to reject the null hypothesis that the residuals did not have circadian or ultradian power.

## Results

### Boundary behavior and bivariate structure of ΔT observations indicates structure in state space

**Fig. 2:**
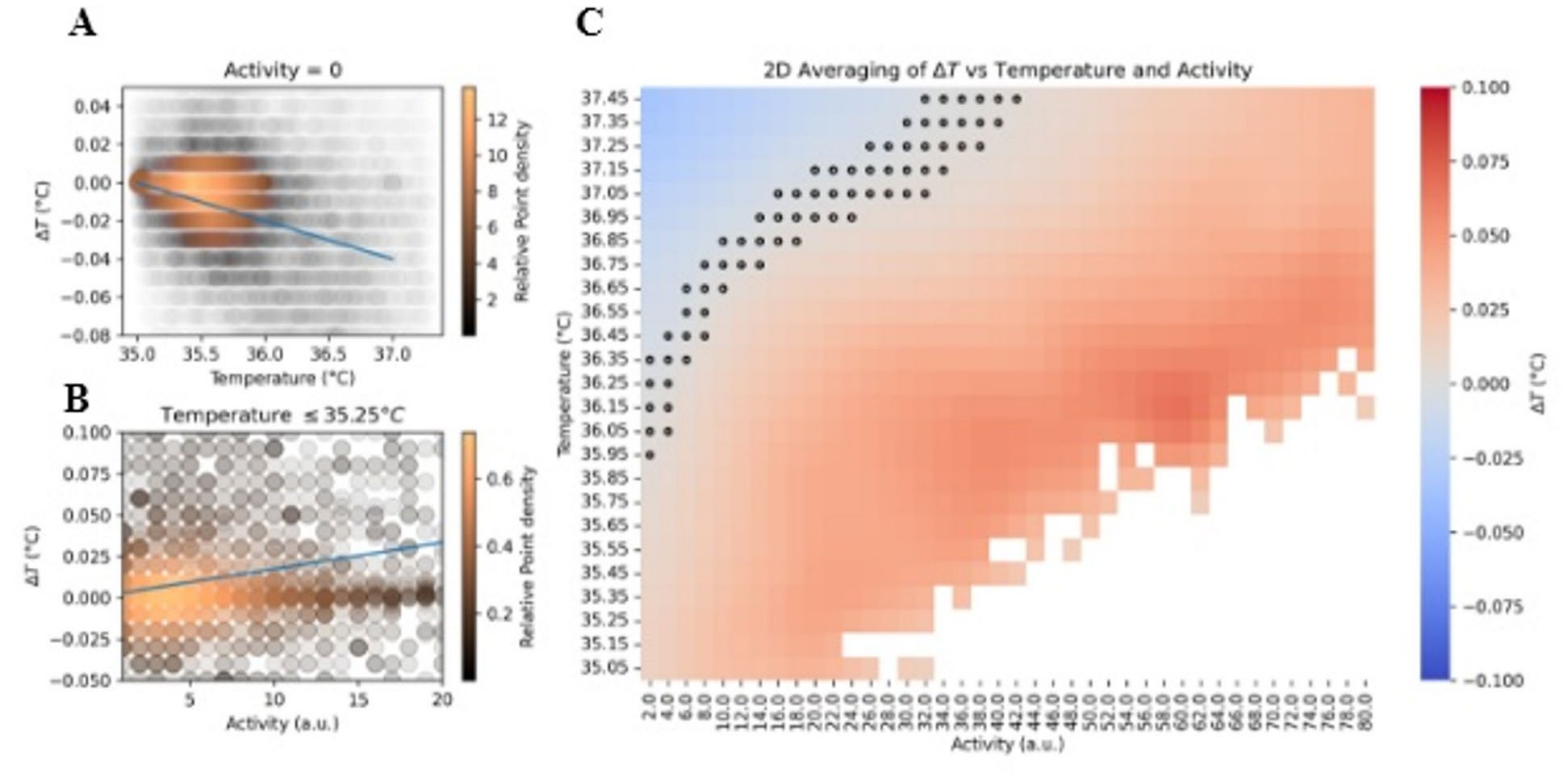
**A**. Scatterplot of the boundary condition CBT and *ΔT* when LA is 0. Color indicates the relative point density estimated using a Gaussian kernel. Brighter (copper) tones represent regions where data points are more densely concentrated. Blue line indicates the optimal linear fit from ordinary least squares. **B**. Scatterplot of the boundary condition LA and *ΔT* when CBT is less than or equal to 35.25 °C. Color indicates the relative point density as in 2 A. Blue line is the optimal linear fit from ordinary least squares. **C**. 2D binned heatmap of the average *ΔT* response in different CBT/LA state variable combinations for all mice. Any cell in which the absolute *ΔT* is less than 0.0025 °C is indicated with a black circle to better state combinations of negligible temperature change. Image generated using the *Python* (3.12.6) package *seaborn* (0.13.2); specifically, *seaborn.heatmap*. Scatterplot of the black circles superimposed on the heatmap were generated using the Python package *matplotlib.pyplot.scatter* (3.9.2).

We first aimed to determine if a state-variable representation of the independent variables, CBT and LA, and the dependent variable, *ΔT*, revealed expected relationships such as a cooling of CBT to steady state relative to current CBT and an LA-induced increase in CBT. To first identify a potential state-dependent linear model, we evaluated the boundary conditions of the two-variable system. A boundary condition describes the behavior of a system when all other variables are at a minimum or maximum value so as to better understand the unique contribution of the variable of interest. This allows us to test whether reach input (CBT or LA) exerts a linear influence on the rate of temperature change, *ΔT*, when the other variable’s effect is negligible. Grouping data from all mice together for an initial sex-generalized model, we first filtered for boundary conditions in which the effects of one of the variables is assumed to be negligible. All *ΔT* vs. CBT in which LA was equal to 0 were selected for the CBT boundary (Fig. 2A), and all *ΔT* vs. LA in which CBT was less than 35.25 °C were selected for the LA boundary (Fig. 2B). Under these boundary assumptions, the full model.

4 $$\:\varDelta\:{T}_{t}={a}_{1}LA-{a}_{2}CBT+C$$

simplifies to single-variable relationships:

5 $$\:\varDelta\:{T}_{t}={a}_{2}CBT+C\, \mathrm{for}\, \mathrm{LA}=0$$

6 $$\Delta\,{T}_t=a_{1}LA+C\, \mathrm{for}\, \mathrm{CBT}<35.25\,^{\circ}{\mathrm{C}}$$

These equations describe how *ΔT* should vary linearly with CBT and LA if the overall system is approximately linear in each dimension. Consistent with this expectation, we found significant linear relationships between CBT and *ΔT* as well as LA and *ΔT* with different directions of effects (Table 1). A higher order polynomial (degree 2) was fit to the boundary conditions as well.

**Table 1:** Linear fits from ordinary least squares analysis of core body temperature (CBT) and locomotor activity (LA) at the boundary conditions.

Variable	Coefficient	Coefficient Value	*P*-Value	95% CI
Core Body Temperature	Constant	0.6963	**< 0.001**	[0.662, 0.730]
	Slope	−0.0199	**< 0.001**	[−0.021, −0.019]
Locomotor Activity	Constant	0.0014	**< 0.001**	[0.001, 0.002]
	Slope	0.0016	**< 0.001**	[0.001, 0.002]

Thus, the boundary condition analysis supports the assumption of state-space linearity. In other words, the rate of temperature change depends linearly on both the current temperature and activity level, consistent with the first-order linear dynamical system described in Eq. 3. This formulation captures the essential thermoregulatory dynamics: a self-correcting decay of CBT toward steady state and a proportional increase in CBT with locomotor activity. Because adding nonlinear terms did not improve predictive accuracy, the linear state-space model provides a parsimonious yet physiologically interpretable description of the system’s behavior.

We then visually evaluated the average *ΔT* response within different bins of CBT and LA to determine if the observed dynamics appeared to change dramatically in bivariate conditions (Fig. 2C). At low LA and higher CBT values cooling dominates. Holding any non-zero LA bin constant within this cooling. However, for the majority of CBT/LA combinations, heating dominates – indicating a general sensitivity of the *ΔT* dynamics to mouse LA.

### Difference equation modeling outperforms linear statistical modeling

**Fig. 3:**
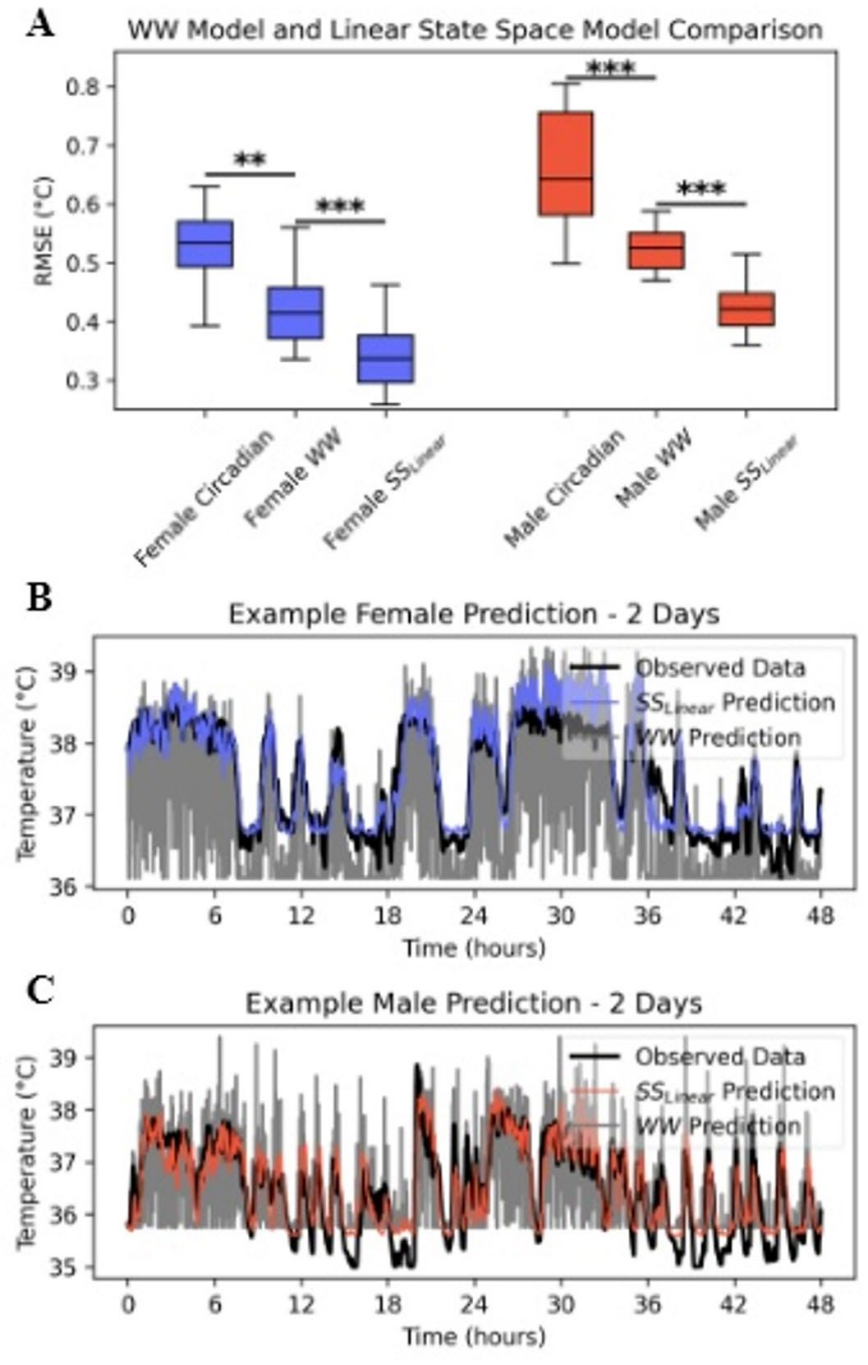
**A**. RMSE of female mouse models (left) and male mouse models (right) (*** *P* < 0.001). Circadian models fit a roughly 24 h sine to the data; the WW Model is the proposed Weinert and Waterhouse Model; the SS_Linear_ model is this work’s proposed dynamical model. **B**. Example of 2880 forecasted CBT samples from 1 starting, observed CBT in a female mouse. WW Model predictions in gray. **C.** Example of 2880 forecasted CBT samples from 1 starting observed CBT in a male mouse. WW Model predictions in gray.

If LA has a heating effect on mouse CBT, and CBT independently has cooling processes to return to steady state, then models which incorporate such causal effects should outperform models that make no assumption of deterministic structure. Such an example can be found in a linear statistical model proposed by Weinert and Waterhouse, 1998 that does not assume temporal structure and instead predicts CBT only from LA. We aimed to test the hypothesis that a linear *state space* model (SS_Linear_) would outperform the prior linear statistical model (Weinert and Waterhouse Model; WW Model) due to its incorporation of cooling effects that may be separate from observed LA magnitudes. We assessed performances with the Root Mean Squared Error (RMSE) of the models’ predictions to the true CBT data.

While the WW Model outperformed a circadian fit mode for both female (*P* < 0.01) and male (*P* < 0.001) mice, the SS_Linear_ model significantly outperformed the WW Model with large effect sizes for paired tests in both the female (*P* < 0.001; paired Cohen’s *d*: 2.1) and male mice (*P* < 0.001; paired Cohen’s *d*: 3.7) (Fig. 3A). Furthermore, visual interrogation of the predictions from both models indicates that the SS_Linear_ model for each mouse much more closely tracks the temporal trajectories of the observed CBT data (Fig. 3B and C). SS_Linear_ coefficients for all mice can be found in Supplementary Table 1.

### Male and female mice are perfectly separable by fitted model parameters

**Fig. 4:**
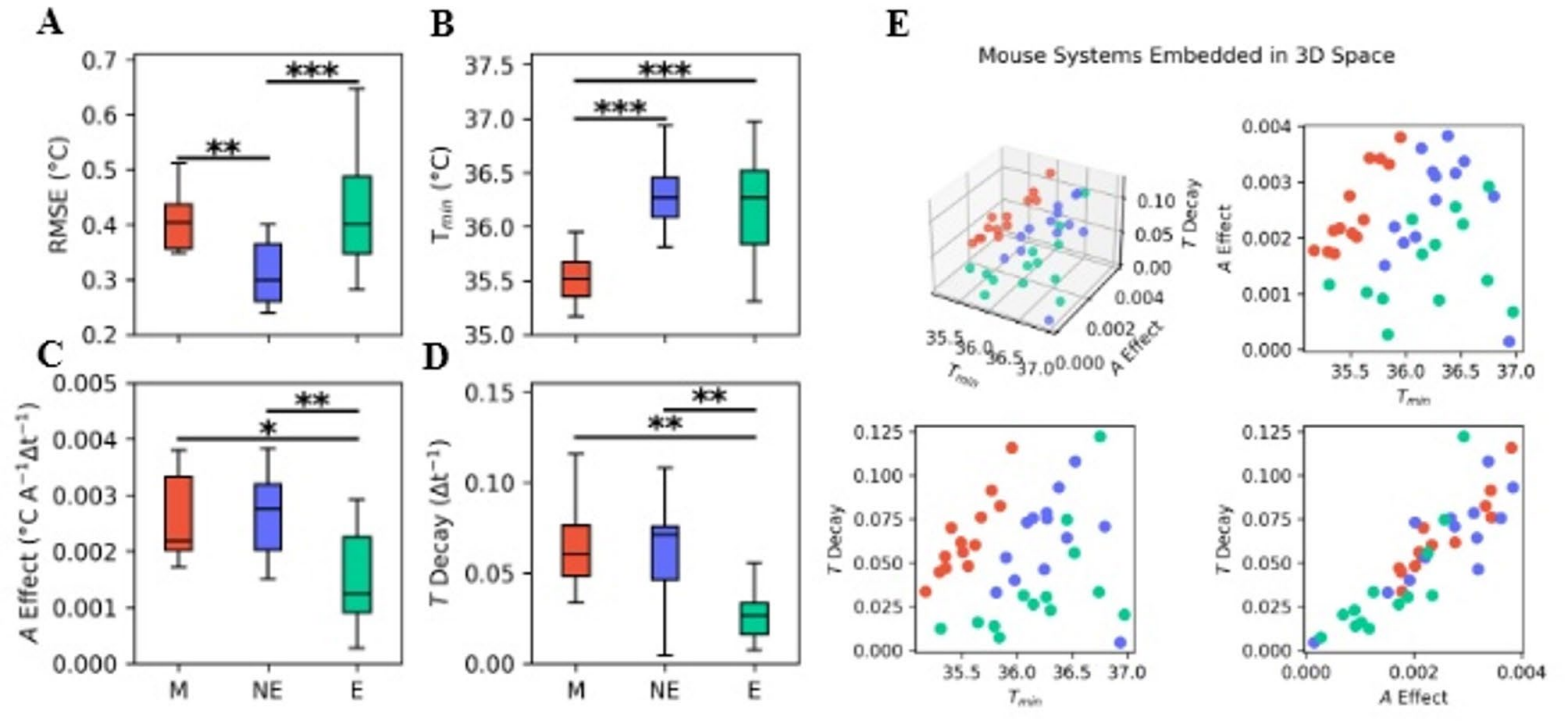
A-D. Boxplot of RMSE, minimum temperature, activity coefficient (A Effect, *a*_*1*_), and temperature decay coefficient (*T* Decay, *a*_*2*_) for male (red), female non-estrus (blue), and female estrus (cyan) mice. **E**. 3D scatterplot of where each mouse falls in the *parameter space* defined by parameter values. Pairwise 2D scatterplots are shown for clarity alongside the 3D scatterplot as lower-dimensional representations of the parameter space.

Females may be harder to fit with a simple model because of the role of hormones in the structure of temperature regulation. Females in estrus may therefore show different parameters than females not in estrus. We confirmed this by first assessing differences in cumulative residual errors (*via* RMSE) between male (M), female non-estrus (NE), and female estrus (E) days. Since we observed significant differences in the RMSE between the types of days (*P* < 0.01) (Fig. 4A), we decided to compare the parameters among the three groups instead of only male/female comparisons to identify potential model differences between sex or estrous phase. Male $\:{T}_{min}$ is significantly lower than both non-estrous days (*P* < 0.001) and estrous days (*P* < 0.001) $\:{T}_{min}$ (Fig. 4B). The estrus LA effect ($\:{a}_{1}$) is significantly lower than both the male (*P* < 0.05) and non-estrus (*P* < 0.01) LA effect (Fig. 4C). The temperature decay effect ($\:{a}_{2}$) is also significantly lower than both the male (*P* < 0.01) and non-estrous (*P* < 0.01) temperature decay effects (Fig. 4D). We plotted the 3-dimensional space defined by the 3 parameters of the linear state space model (Fig. 4E) and observed that the male and female samples (red and blue/cyan, respectively) could be perfectly separated with a simple curve.

### Residuals of the linear state space model reveal time-varying temperature dynamics based on sex and estrous

**Fig. 5:**
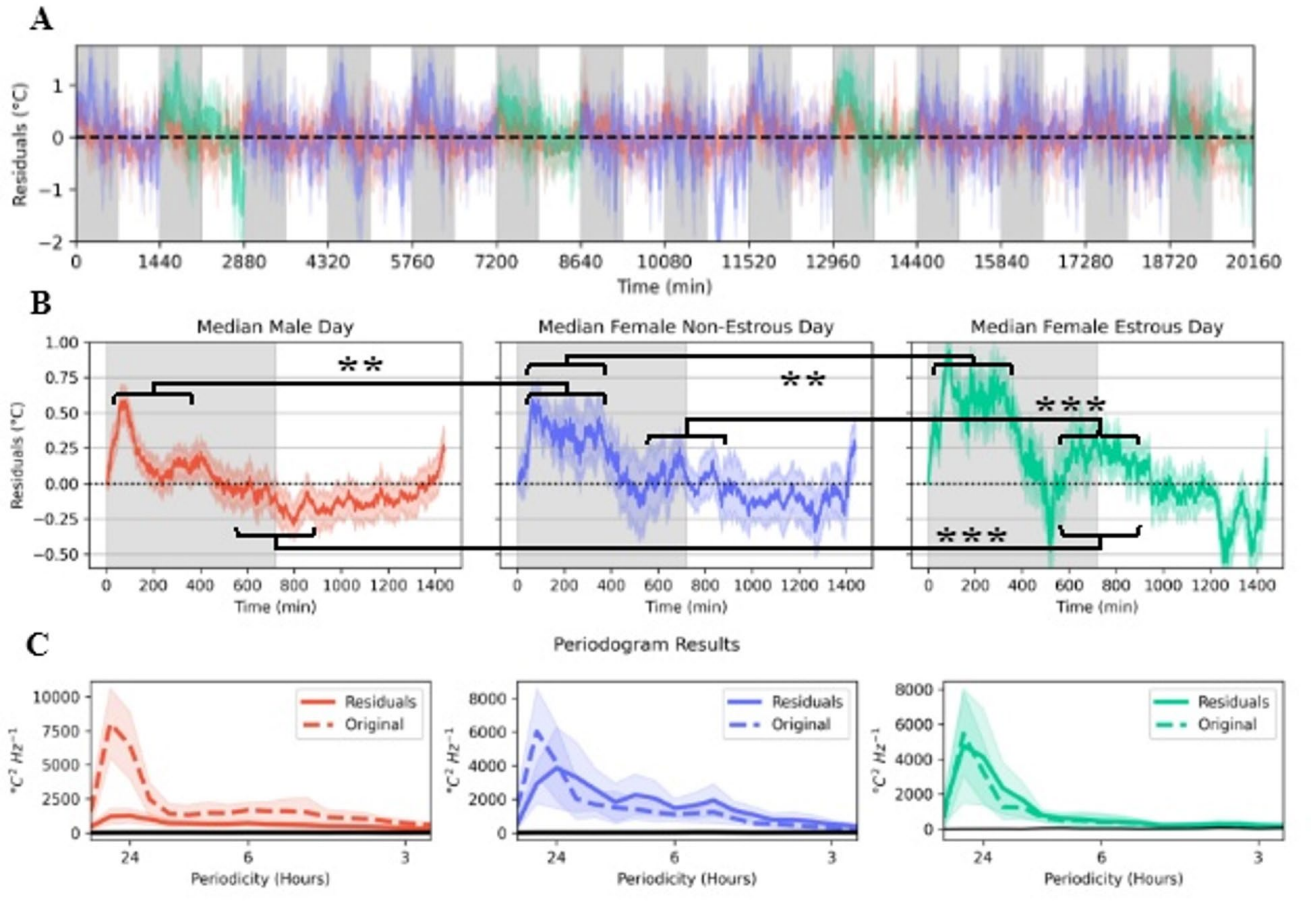
**A**. Average residuals of the 14 days of CBT data when differencing out the predicted effects of activity and thermoregulation. Red, blue, and cyan represent residuals from male mice, female non-estrous days, and female estrous days, respectively. **B**. The average day of residuals for each type of day (male: red, non-estrous: blue, estrous: cyan) are overlaid on each other, with the mean day in solid color. **C**. Solid line: mean Welch’s periodogram for each group of days. The ‘Residuals’ line is generated from the periodogram of the residual data in the time domain after removing the linear state space predictions. The ‘Original’ line is generated from the periodogram of the *observed* CBT data without any sort of filtering. Shaded region: standard deviation of each group’s periodograms. Solid black line is the 95th percentile of the null hypothesis that the analyzed signal is Gaussian white noise. (**P < 0.01, ***P < 0.001).

The proposed LA-CBT linear state space model of CBT is meant to account for the aperiodic, unaligned effects of LA as well as the expected regulation of CBT back to estimated $\:{T}_{min}$. By removing the drift, or deterministic structure, from the observed CBT values we are left with the sequential (or temporal) residuals. If it were the case that the linear state space model can nearly predict CBT values (barring measurement error), then we would expect the sequential residuals to have a constant variance and mean of 0. Sequential residuals that do not meet these criteria may have predictable/deterministic structure that the model has not accounted for. Deterministic structure can be inferred if there is serial correlation in the residuals – that is, there are significant lags in the autocorrelation plot of the residuals. We observe such serial correlations in all mice that can be visibly observed from the residual’s plots (Fig. 5A; Durbin-Watson test statistics for serial correlation of residuals for each mouse can be found in **Supplementary Fig. 1**). Positive and negative sequential residuals indicate the model underestimated ($\:{T}_{t}-{\widehat{y}}_{t}>0$) and overestimated ($\:{T}_{t}-{\widehat{y}}_{t}<0$), respectively.

The population mean of the sequential residuals are grouped by male (red), female non-estrus (blue), and female estrus (cyan). We took the average 24-hr (start of lights off to start of lights off the following day) period of sequential residuals for each group (Fig. 5B) and evaluated if there were periods of time wherein the linear state space model had different prediction performances. The estrus group had a significantly greater sum of sequential residuals than both the male (Mann-Whitney U; *P* < 0.01) and the non-estrous (Mann-Whitney U, *P* < 0.01) groups between 1 h and 6 h post-lights on, as well as a greater sum of sequential residuals than both the male (Mann-Whitney U, *P* < 0.001) and non-estrous (Mann-Whitney U, *P* < 0.001) between 10 and 14 h post-lights on.

If it were the case that the within-day sequential residuals do not contain any periodicity, then we would observe no significant differences between the magnitudes in the mean periodograms across all mice (while accounting for the kind of day – male, non-estrus, and estrus) and a random shuffling of the original data (in brief, shuffling a timeseries can break potential cyclic structure). However, we reject this null hypothesis in all three instances of the sequential residuals (Fig. 5C).

## Discussion

Here we designed a 3-parameter, state space model of LA’s impact on CBT, as well as CBT’s cooling dynamics, that achieved high performance with stability at long time horizons. By using minute-level data, the within-mouse parameters could be fit to identify each individual’s sex or estrous state. Finally, the model allowed for the removal of deterministic physiological variance (e.g., LA and cooling) from CBT, which resulted in the sequential residual CBT signal in which we found circadian power in CBT from non-LA related causes. This supports the study of the influence of other factors besides LA and innate cooling on temperature, such as circadian, ultradian, and infradian timescales of hormonal change where LA’s interference might otherwise make such analyses impossible.

There is strong evidence for the existence of infradian, circadian, and ultradian rhythms in mice across many biochemical and physiological variables^2,13,15–17^. With intraperitoneal physiological sensors, signals such as core CBT) and LA can be sampled at high temporal resolutions. Various rhythms in these variables can then be compared across different murine states, conditions, or interventions. However, the dynamics of CBT oscillations are not necessarily driven by the linear summation of multiple underlying rhythms. The observed dynamics in CBT are driven by numerous underlying variables such as deterministic cooling processes, aperiodic metabolic activity, quasiperiodic as well as periodic hormonal effects, and their causal interactions therein. These numerous interacting components make it difficult to isolate any singular contribution to CBT. To accurately identify the relationship between CBT and any of these other variables (independent of the causal effects of the other underlying variables), a model must be designed to disentangle subsets of the interactions that give rise to the global dynamics. In this work, we proposed a deterministic mathematical model of CBT, independent of time as a variable, that incorporates the state-dependent effects of innate cooling of CBT and the aperiodic heating effects from LA. The model terms are interpretable, parsimonious, and generalizable to multiple mice. We show that there are sex- and estrous-dependent differences in the global errors and coefficients/parameters when fitting the model terms to each mouse. Furthermore, we show that the high-dimensional projection of each mouse in their parameter-space separates male from female mice with 100% accuracy. We then show the long-term stability of forecasting 20159 samples for each mouse using only their *first observed CBT* as well as their minute-level LA. The minute-level LA is revealed to contribute power to the circadian *and* ultradian frequency bands due to its aperiodic structure combined with the subsequent decay in CBT from its current value. Nonetheless, significant circadian and ultradian spectral power is still present in the residual data for the mice even after differencing out the causal effects of CBT cooling and LA heating – indicating leftover endogenous chronobiological mechanisms of thermal control.

While there are multiple ways to model physiological timeseries, there are several reasons why we chose a mathematical framework over a statistical (e.g., autoregressive with exogenous variables) or machine learning (e.g., neural network) framework. Primarily, we hypothesized that the *coefficients/parameters* of a process carry valuable information about the underlying dynamics of physiological timeseries. Our proposed 3-parameter, state space model conveys information about the global dynamical properties of the CBT timeseries based only on only the most recent predicted CBT amplitude and measured actigraphy. The coefficients/parameters therefore have units that convert these variables into the expected rate of change of CBT over a window of time - $\:{\varDelta\:t}^{-1}$ and $\:^\circ\:C{{A}^{-1}\varDelta\:t}^{-1}$ for temperature cooling and LA effects, respectively. These useful interpretations of coefficients are lost when considering the statistical models that incorporate values from further in the past as well as the neural network models that obfuscate the parameters and variables at deeper layers. The mathematical framework allows us to directly compare *different models* to each other, which makes possible the separation of unique underlying physiological states based on sex and estrous. Furthermore, the primary endpoint of the mathematical representation is not error minimization. Error minimization is necessary for model validation but is not sufficient for system interpretation. A neural network would attempt to reduce the residuals to gaussian white noise, and a complex enough model would likely outperform the RMSE of the mathematical model. However, the residual data after differencing out the deterministic, expected variance of the interactions between LA and CBT reveal underlying structure in the data that may be related to underling endogenous chronobiological mechanisms. This is information one would want to retain when studying such processes, but which would be lost with a purely error-minimizing black box.

Although the identification of endogenous chronobiological mechanisms (gene regulation, hormones, etc.) is certainly not new, there are opportunities to quantify the underlying causal relationships between such mechanisms and complex multivariate signals like CBT. Understanding the *forward* direction of how these mechanisms impact CBT provides intuition for the *reverse* direction of how CBT dynamics may reveal changes in underlying mechanisms or states (sex differences, estrous, etc.). However, there is reduced confidence in inferring the reverse direction when there is a plethora of endogenous and exogenous signals that drive changes in CBT. A benefit of developing mathematical models of temporal data is that both the residual data *as well as* the fit parameters of the models provide unique information regarding the underlying dynamics of physiological processes. We believe that the CBT model described in this work is an intuitive starting point for removing causal LA effects and deterministic CBT cooling effects from longitudinal CBT data. We also believe that the separation of CBT and LA effects is the reason why the linear state space model outperformed the prior WW Model. More specifically, the WW Model assumes that a rolling sum of the magnitude of LA describes the same unit change in $\:\varDelta\:T$ whether LA increases are decreases due to the equal weighting of the $\:\tau\:$ previous samples. However, since we propose in our linear state space model that LA only has *positive* effects on $\:\varDelta\:T$, this allows for linearly separable dynamics in which CBT’s distance from a minimum value ($\:{T}_{min})$ incorporates the *negative* pressure for CBT to return to steady state. As such, our proposed model disentangles the two separate heating and cooling processes that would be integrated together if using the WW Model.

We developed this model using data collected for other purposes, and so prospective data gathering could be aimed at expanding the number of causal factors such models might account for. The following limitations suggest themselves for such efforts: (1) because the mice were allowed to feed *ad libitum*, the aperiodic thermal effects of food intake could not be accounted for; (2) information regarding mouse size (e.g., BMI) was not recorded – size likely affects the decay constant of CBT (more mass at a certain temperature is able to buffer cooling effects) as well as LA’s causal effect on CBT (i.e., more energy is needed per unit actigraphy to move more mass); (3) darkness/light-seeking behavior was not accounted for, which may in turn affect local ambient temperature depending on mouse location (however, we believe this effect would be smaller in magnitude compared to the prior ones listed); (4) we assumed the model parameters themselves are constant – due to physiological parameters likely reflecting other underlying physiological variables, there is no reason to believe that these parameters should be stationary through time.

Despite these limitations, we believe the model in its current form could be used in studies involving the modification of gene expression and/or hormones that may lead to secondary effects in CBT dynamics^11,12,22–25^. The model serves as a tool for removing expected cooling effects in CBT as well as causal heating effects of LA, which we believe is a powerful method of denoising experiments in which CBT is an explicit statistical endpoint of some intervention or experiment. The value of this model is made further apparent when considering any intervention that may lead to changes in LA, which would further drive changes in CBT dynamics, and so occlude the ability to study the CBT response patterns (as opposed to LA effects). We hope that the publishing of this model within an easy-to-use *Python* library will encourage its further use and development in murine studies where the disentangling of causal interactions on CBT dynamics is of interest.

## Supplementary Information

Below is the link to the electronic supplementary material.

Supplementary Material 1

Supplementary Material 2

## Data Availability

Data is made freely available in the supplemental materials. Individuals that use the data for future work should cite Smarr, 2017^20^.

## Code availability

Code will be made freely available in the supplemental material upon acceptance of this work for publication.
