# Supplementary material for: Dynamical model of aperiodic locomotor activity effects on mouse core body temperature removes transient perturbations from longitudinal temperature signals
Source: Sci Rep. 2026 Jan 6;16:2164. doi: 10.1038/s41598-025-31953-8 (PMC12808673; doi:10.1038/s41598-025-31953-8)
Supplement: Supplementary file 2 — Supplementary Material 2 [file 41598_2025_31953_MOESM2_ESM.docx]

**Supplementary Table 1**. Parameters for each mouse using the Linear State Space model.

| **Mouse ID** | **Coefficients from Estrus?** | **T_min** | *A* Effect (a_1) | *T* Decay (a_2) |
| --- | --- | --- | --- | --- |
| F1 | No | 36.24541 | 0.003192 | 0.046318 |
|  | Yes | 36.26706 | 0.001884 | 0.03043 |
| F2 | No | 36.52929 | 0.003376 | 0.108099 |
|  | Yes | 36.75157 | 0.002925 | 0.122323 |
| F3 | No | 36.14244 | 0.00361 | 0.075774 |
|  | Yes | 36.05982 | 0.002342 | 0.031385 |
| F4 | No | 36.38159 | 0.003838 | 0.093231 |
|  | Yes | 36.52007 | 0.002256 | 0.055725 |
| F5 | No | 36.79625 | 0.002752 | 0.070889 |
|  | Yes | 36.74076 | 0.001243 | 0.033313 |
| F6 | No | 36.27347 | 0.002682 | 0.075326 |
|  | Yes | 36.30259 | 0.000895 | 0.023071 |
| F7 | No | 36.45243 | 0.003165 | 0.064358 |
|  | Yes | 36.14861 | 0.001712 | 0.026451 |
| F8 | No | 36.27024 | 0.003108 | 0.078484 |
|  | Yes | 36.45122 | 0.002566 | 0.074821 |
| F9 | No | 36.08881 | 0.002021 | 0.0732 |
|  | Yes | 35.83849 | 0.000275 | 0.007369 |
| F10 | No | 35.98063 | 0.001915 | 0.040011 |
|  | Yes | 35.64458 | 0.001023 | 0.016056 |
| F11 | No | 36.93816 | 0.000147 | 0.004618 |
|  | Yes | 36.974 | 0.000683 | 0.020286 |
| F12 | No | 35.898 | 0.002203 | 0.052959 |
|  | Yes | 35.79215 | 0.000911 | 0.013854 |
| F13 | No | 35.81201 | 0.001509 | 0.033067 |
|  | Yes | 35.3081 | 0.001171 | 0.012579 |
| M1 | N/A | 35.67344 | 0.003441 | 0.076197 |
| M2 | N/A | 35.77063 | 0.00342 | 0.091543 |
| M3 | N/A | 35.34506 | 0.002137 | 0.053796 |
| M4 | N/A | 35.40327 | 0.002178 | 0.070205 |
| M5 | N/A | 35.62119 | 0.002329 | 0.060265 |
| M6 | N/A | 35.35228 | 0.001718 | 0.04708 |
| M7 | N/A | 35.17 | 0.001778 | 0.033701 |
| M8 | N/A | 35.84693 | 0.003328 | 0.082706 |
| M9 | N/A | 35.2966 | 0.00176 | 0.044856 |
| M10 | N/A | 35.49004 | 0.002757 | 0.06163 |
| M11 | N/A | 35.55604 | 0.002018 | 0.048241 |
| M12 | N/A | 35.51205 | 0.002093 | 0.056185 |
| M13 | N/A | 35.95275 | 0.003807 | 0.115911 |


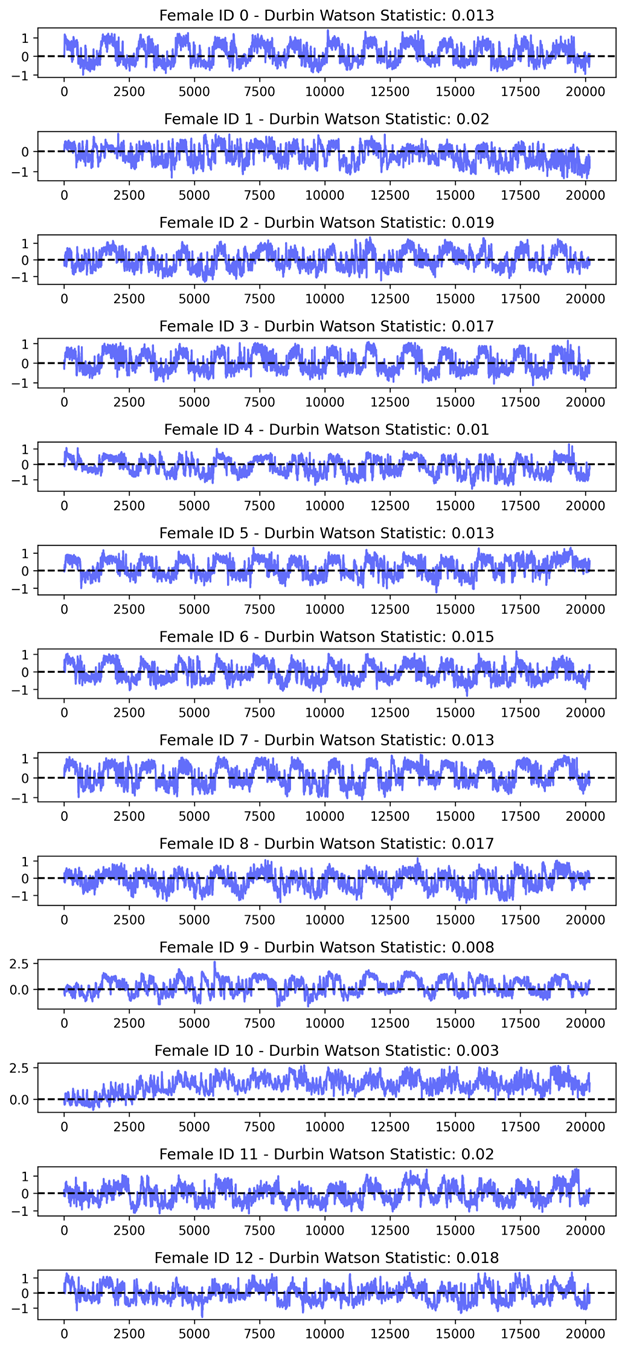

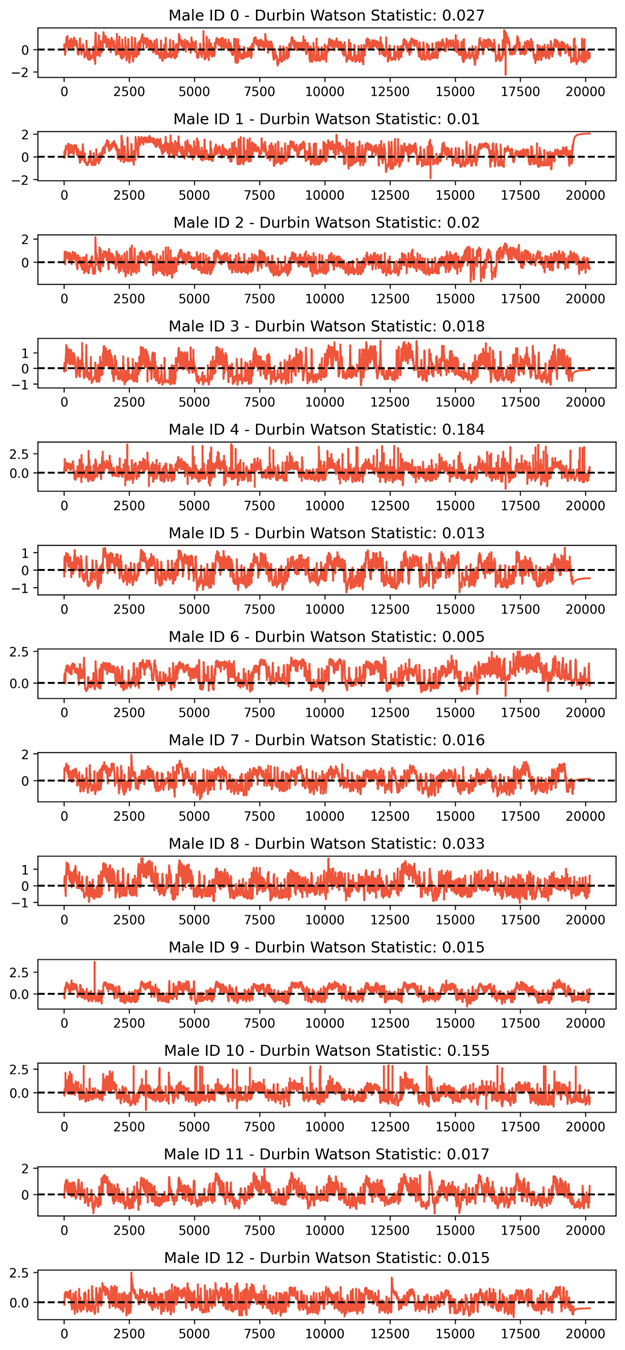


**Supplementary Figure 1**. **Left**. Residuals after fitting a linear state space model to each female mouse. Durbin Watson test statistics are placed in the titles for each subplot. **Right**. Residuals after fitting a linear state space model to each male mouse. Durbin Watson test statistics are placed in the titles for each subplot. Durbin Watson test statistics close to 0 indicate strong positive autocorrelation of residuals. Test statistics close to 2 indicate no autocorrelation of residuals. Values close to 4 indicate strong negative autocorrelation of residuals.
